# Supplementary material for: A warm-white light-emitting diode based on single-component emitter aromatic carbon nitride
Source: Nat Commun. 2022 Oct 30;13:6495. doi: 10.1038/s41467-022-34291-9 (PMC9618563; doi:10.1038/s41467-022-34291-9)
Supplement: Supplementary file 1 — Supplementary Information [file 41467_2022_34291_MOESM1_ESM.pdf]

Supplementary Information

A Warm-White Light-Emitting Diode Based on Single-Component Emitter Aromatic Carbon Nitride

Yunhu Wang[1], Kunpeng Wang[1], Fangxu Dai[1], Kai Zhang[1], Haifeng Tang[1], Lei Wang[1,2]*, Jun Xing[1,]*

[1]Key Laboratory of Eco-chemical Engineering, Ministry of Education, College of Chemistry and Molecular Engineering, Qingdao University of Science & Technology, Qingdao 266042, China.

[2]Shandong Engineering Research Center for Marine Environment Corrosion and Safety Protection, College of Environment and Safety Engineering, Qingdao University of Science and Technology, Qingdao 266042, China.

*Correspondence: inorchemwl@126.com (L.W.), xingjun@qust.edu.cn (J.X.)

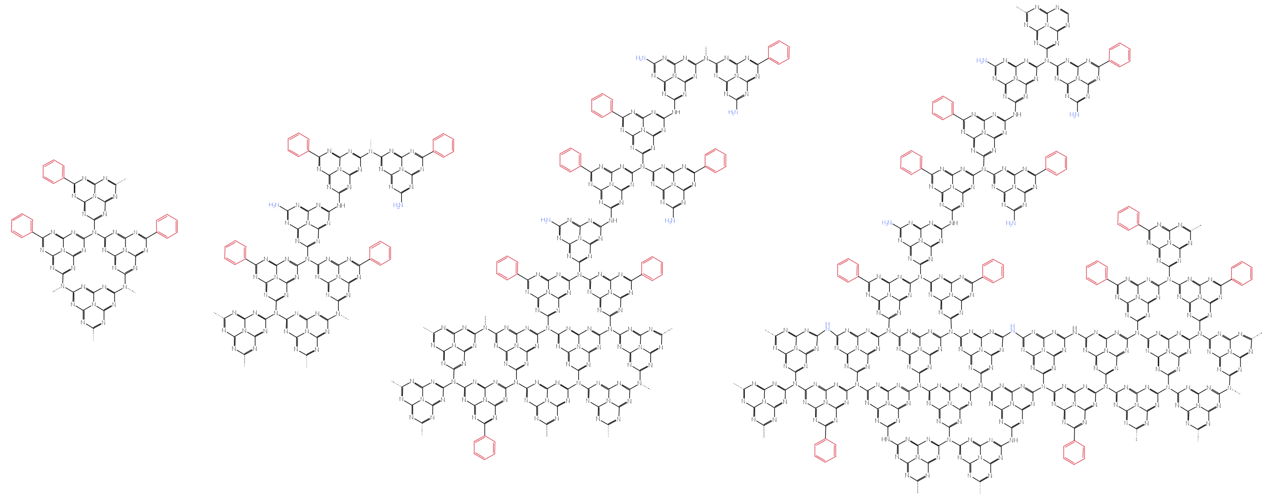

C/N=1.48 C/N=1.27 C/N=1.15 C/N=1.08

Supplementary Note 1 Schematic diagram of PhCN with different polymerization degrees. The C/N moles of PhCN models with different degrees of polymerization are shown in the figure above. As the degree of polymerization increases, the C/N mole ratio of PhCN model decreases gradually.

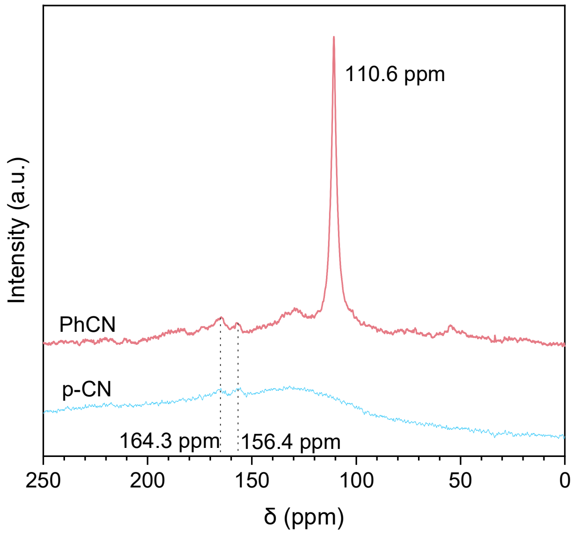

Supplementary Fig. 1 Solid-state ^{13}C NMR spectra of p-CN and PhCN powders.

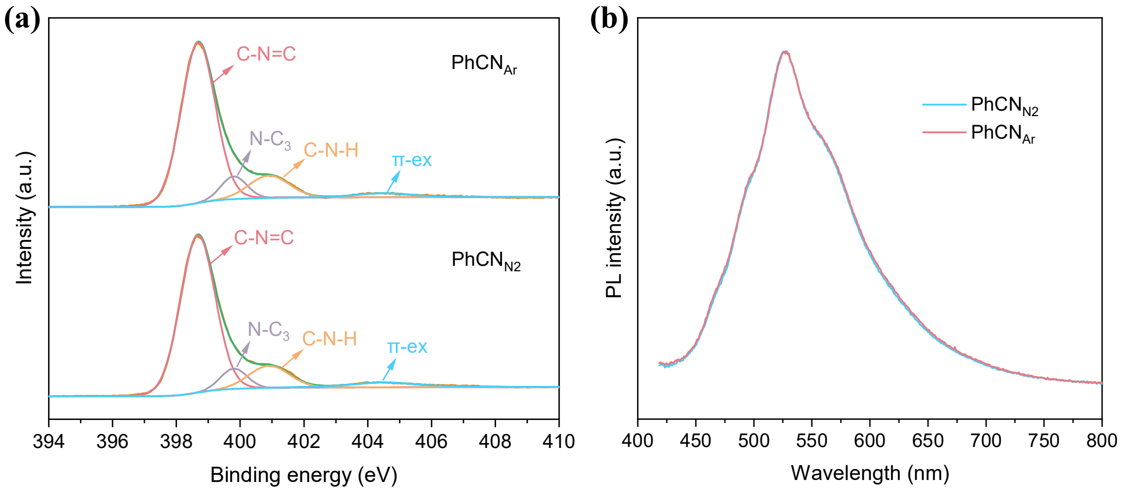

Supplementary Fig. 2 The properties of PhCN powders prepared under different atmospheres. (a) XPS N 1s spectra and (b) PL spectra of PhCN powders synthesized at N_2 and Ar atmosphere. The N 1s spectra of both samples are nearly the same and contain four peaks at 398.6, 399.8, 400.9, and 404.5 eV, which are corresponding to sp^2-bonded N in triazine rings (C=N-C), tertiary nitrogen bonded to carbon atoms (N-C_3), amino groups (C-N-H) and charging effects or positive charges localization in the heterocycles (π-excitations), respectively. There is no difference in PL spectra between $PhCN_{Ar}$ and $PhCN_{N2}$, and no significant variation in PLQY (38% vs 40%).

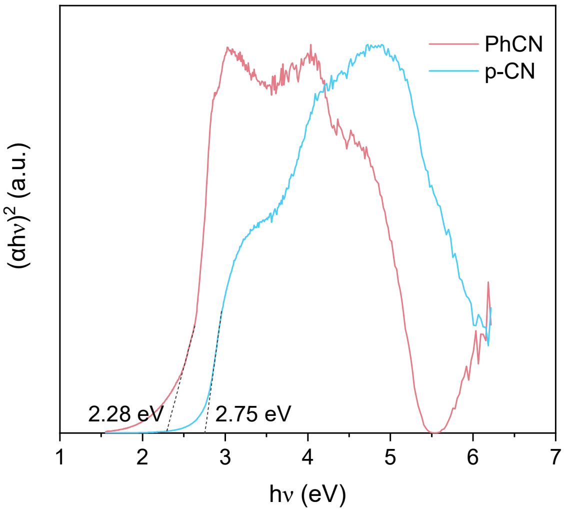

Supplementary Fig. 3 The bandgaps of g-CN materials. Tauc plots of p-CN and PhCN. The bandgap width of g-CN material is calculated by using the following formula:

$$(\alpha h\upsilon)^{\frac{1}{n}} = \mathrm{A}(h\upsilon - E_{\mathrm{g}}) \qquad (1)$$

Where α, h, υ, and E_{g}, are absorption coefficient, Planck constant, light frequency, and bandgap, respectively. For direct bandgap semiconductors, the n value is $\frac{1}{2}$.

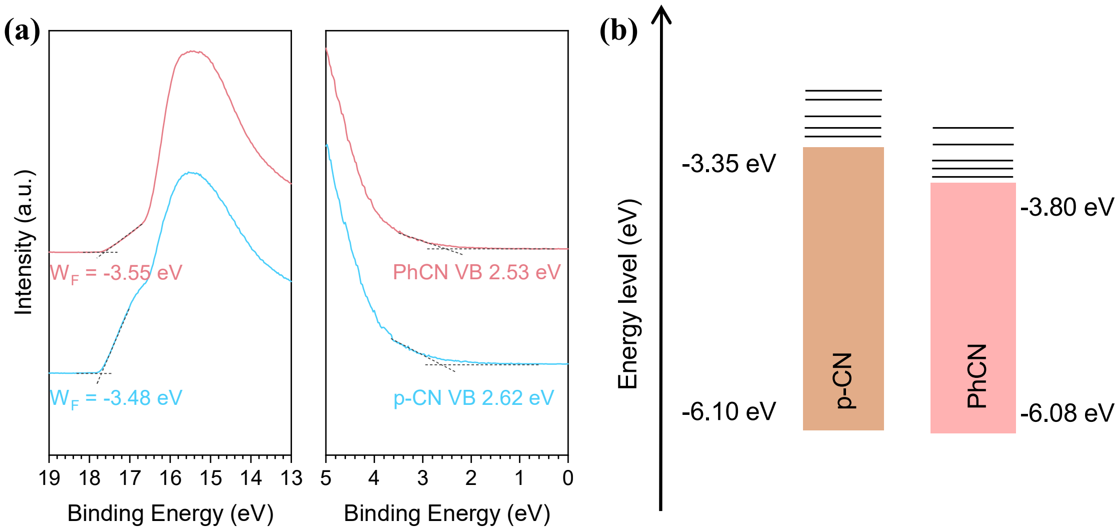

Supplementary Fig. 4 The energy levels of g-CN materials. (a) Ultraviolet photoelectron spectroscopy (UPS) test results and (b) energy level diagrams of p-CN and PhCN (The black line represents LUMO+1, LUMO+2, ……LUMO+*n* energy level).

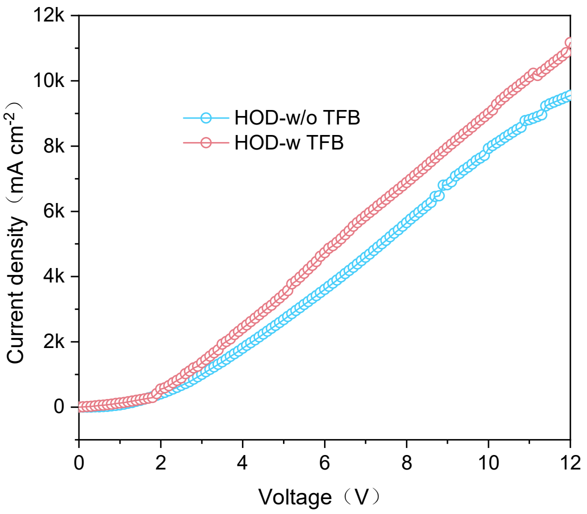

Supplementary Fig. 5 Current density-voltage curves of hole-only devices (HOD). HOD structure: ITO/PEDOT:PSS/with or without TFB/PVK/PhCN (5 nm)/MoO_3 (40 nm)/LiF (1 nm)/Al (80 nm).

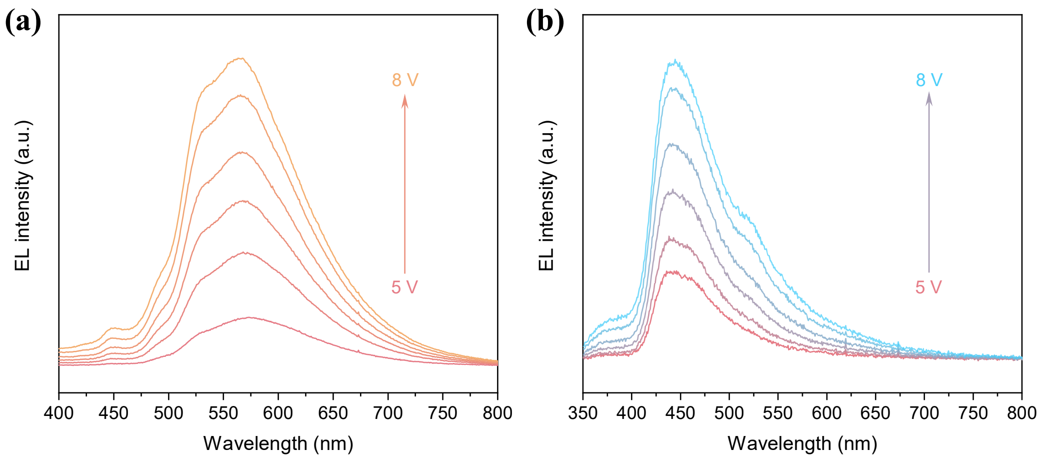

Supplementary Fig. 6 EL spectra of LEDs. The EL spectra of (a) device II and (b) device IIc under 5-8 V.

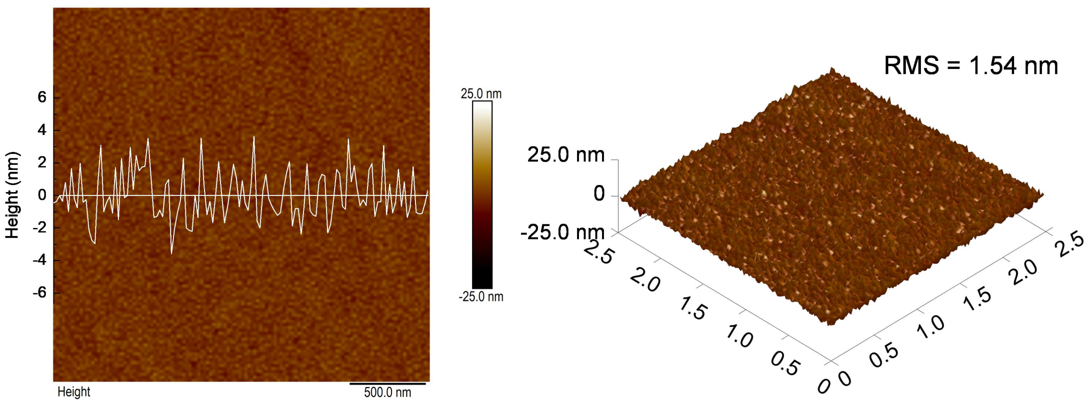

Supplementary Fig. 7 AFM images of p-CN films deposited on PEDOT:PSS/TFB/PVK surfaces. Height images (left), corresponding line-scan profile images and pseudo-three-dimensional images (right).

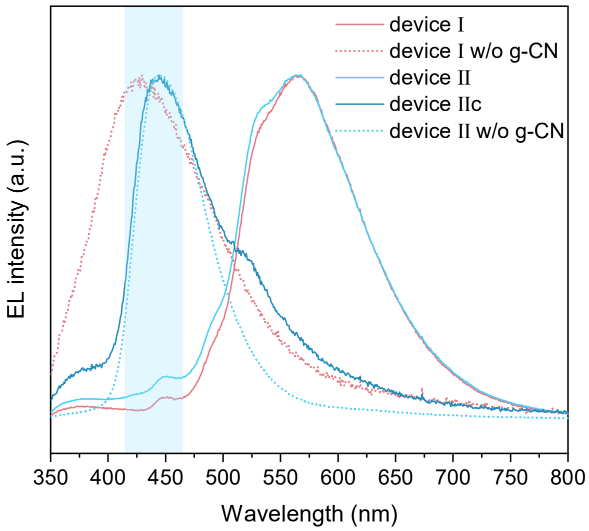

Supplementary Fig. 8 EL spectra of devices I and II with or without g-CN materials. Device I w/o g-CN structure: ITO/PEDOT:PSS/PVK/TPBi (40 nm)/LiF (1 nm)/Al (80 nm), device II w/o g-CN structure: ITO/PEDOT:PSS/TFB/PVK/TPBi (40 nm)/LiF (1 nm)/Al (80 nm).

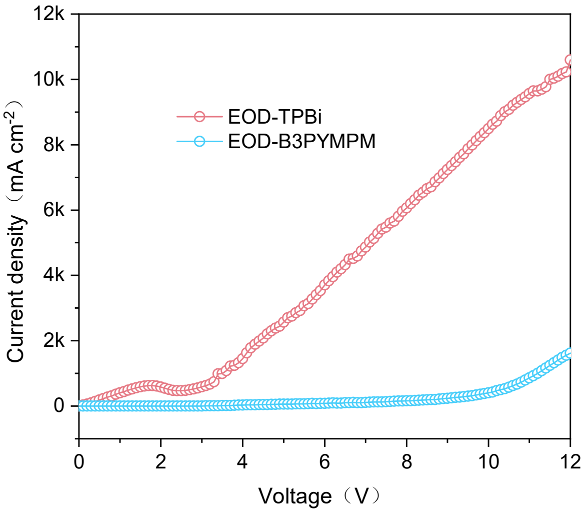

Supplementary Fig. 9 Current density-voltage curves of electronic-only devices (EOD). EOD-TPBi structure: ITO/TPBi (20 nm)/PhCN (5 nm)/TPBi (20 nm)/LiF (1 nm)/Al (80 nm), EOD-B3PYMPM structure: ITO/B3PYMPM (20 nm)/PhCN (5 nm)/B3PYMPM (20 nm)/LiF (1 nm)/Al (80 nm).

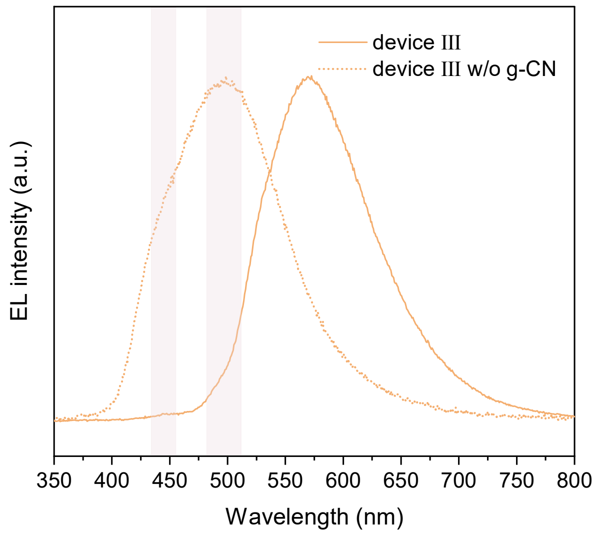

Supplementary Fig. 10 EL spectra of device III with or without g-CN materials. Device III w/o g-CN structure: ITO/PEDOT:PSS/TFB/PVK/B3PYMPM (40 nm)/LiF (1 nm)/Al (80 nm).

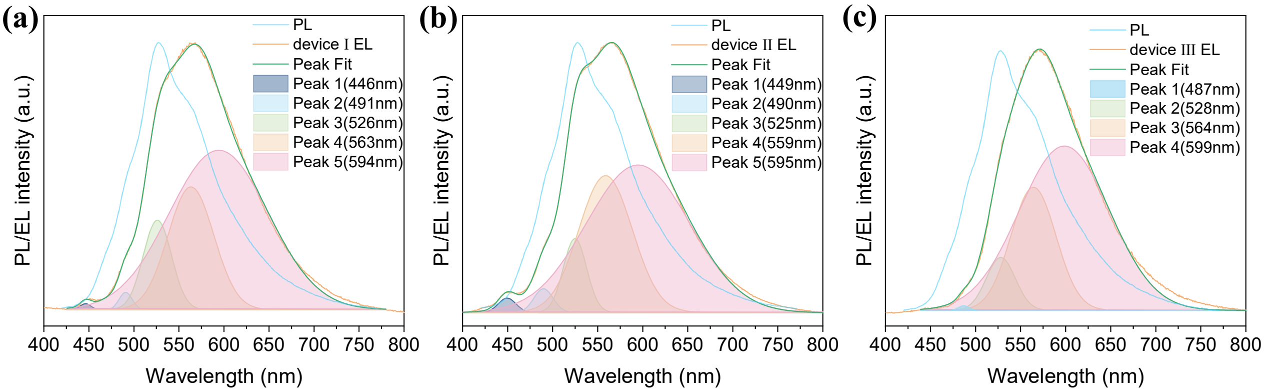

Supplementary Fig. 11 EL spectra fitting. The EL spectra and corresponding peak fitting results of device I (a), II (b) and III (c).

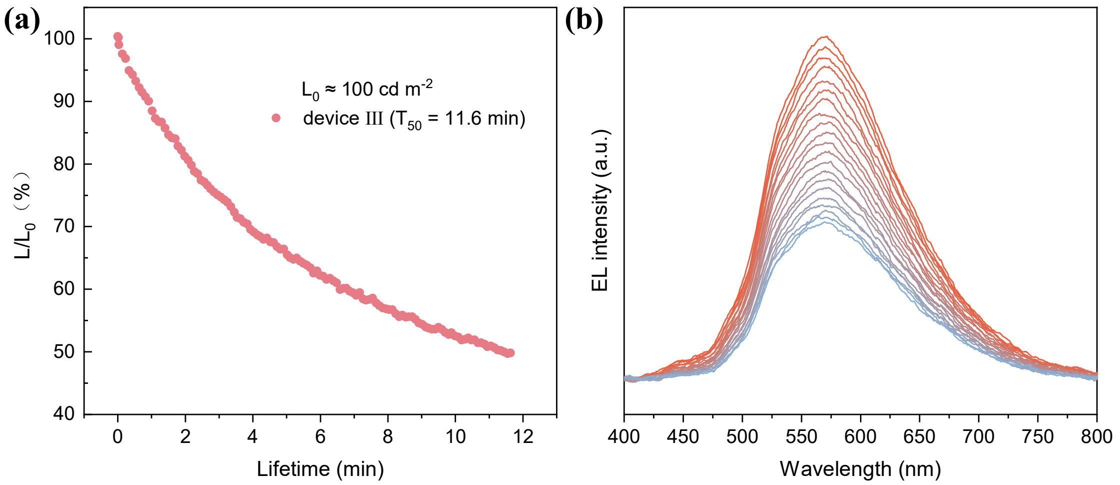

Supplementary Fig. 12 Operational lifetime and spectral stability test of LEDs. (a) Device operational lifetime measured in constant current mode and (b) the corresponding spectral stability of device III.

Supplementary Table 1 Elemental analysis results of the p-CN and PhCN samples.

Sample	C (wt.%)	N (wt.%)	H (wt.%)	C/N (atom%)
p-CN	35.93	53.82	2.342	0.778
$PhCN_{N2}$	45.57	48.08	2.507	1.105
$PhCN_{Ar}$	45.51	48.29	2.516	1.100

Supplementary Table 2 PL fitting data of the PhCN sample.

Peak Fit	Peak center (nm)	Peak area	Peak area percentage (%)
Peak 1	470	3.24	2.93
Peak 2	490	5.16	4.68
Peak 3	524	32.95	29.83
Peak 4	565	34.88	31.58
Peak 5	588	34.22	30.98

Supplementary Table 3 PL lifetime of the p-CN and PhCN samples.

Sample	emission wavelength (nm)	τ_1 (ns)	τ_2 (ns)	τ_3 (ns)	τ_{ave} (ns)
p-CN	465	1.30	4.59	23.07	4.36
PhCN	470	0.82	5.36	43.28	4.06
	490	0.98	6.35	60.50	6.11
	525	4.36	77.71	-	18.70
	565	4.52	86.37	-	22.37
	600	3.41	79.07	-	11.19
