# Supplementary material for: A warm-white light-emitting diode based on single-component emitter aromatic carbon nitride
Source: Nat Commun. 2022 Oct 30;13:6495. doi: 10.1038/s41467-022-34291-9 (PMC9618563; doi:10.1038/s41467-022-34291-9)
Supplement: Supplementary file 3 — Description of Additional Supplementary Files [file 41467_2022_34291_MOESM3_ESM.pdf]

Supplementary Video 1 The operation of the LED device III.
